# Do Purpose in Life and Social Support Mediate the Association between Religiousness/Spirituality and Mortality? Evidence from the MIDUS National Sample

**DOI:** 10.3390/ijerph20126112

**Published:** 2023-06-13

**Authors:** Jennifer Morozink Boylan, Christianne Biggane, Jonathan A. Shaffer, Caitlyn L. Wilson, Kaitlyn M. Vagnini, Kevin S. Masters

**Affiliations:** 1 Department of Health and Behavioral Sciences, University of Colorado Denver, Denver, CO 80217, USA; 2 School of Medicine, University of Colorado, Aurora, CO 80045, USA; christianne.biggane@cuanschutz.edu; 3 Department of Psychology, University of Colorado Denver, Denver, CO 80217, USA; jonathan.shaffer@ucdenver.edu (J.A.S.); caitlyn.wilson@ucdenver.edu (C.L.W.); kaitlyn.vagnini@ucdenver.edu (K.M.V.); kevin.masters@ucdenver.edu (K.S.M.)

**Keywords:** religion, spirituality, mortality, purpose in life, social support, midlife, older adults

## Abstract

We examined prospective associations between religiousness/spirituality (R/S; i.e., service attendance, R/S identity, R/S coping, spirituality) and all-cause mortality in the Midlife in the United States (MIDUS) sample, including whether having a purpose in life and positive social support are indirect pathways through which R/S predicts mortality. We examined service attendance and a composite of R/S identity, R/S coping, and spirituality from the baseline wave (1995–1996; n = 6120 with complete data), purpose in life and positive social support from the second wave (2004–2006), and vital status through 2020 (n = 1711 decedents). Cox regression models showed that attending religious services more than weekly and approximately weekly was associated with a lower mortality risk compared to never attending in the adjusted models (>weekly vs. never, HR (95% CI) = 0.72 (0.61, 0.85); weekly vs. never, HR (95% CI) = 0.76 (0.66, 0.88)). The R/S composite was also associated with lower mortality risk in the adjusted models (HR (95% CI) = 0.92 (0.87, 0.97)). Indirect effects from R/S to mortality via purpose in life and positive social support were significantly different from zero. These findings highlight the importance of multidimensional aspects of R/S for population health and point to purpose in life and positive social support as underlying pathways between R/S and mortality.

## 1. Introduction

Religion and spirituality are important in the lives of many. Religious and spiritual beliefs affect behaviors, cognitions, and emotions and can fundamentally shape one’s worldview and actions therein [1]. Results from the 2022 Gallup poll show that 78% of Americans report a religious affiliation and 46% report that religion is “very important” in their lives [2]. Despite religious affiliation and importance declining in the U.S. in recent decades, 81% report believing in God, and 75% report praying to God outside of religious services “often” or “sometimes” in the 2022 Gallup data [2]. Across 15 countries in Western Europe in 2017, 71% of the population identified as Christian, although only 22% reported attending religious services at least monthly [3]. Further, more than 70% of the populations in many countries in Africa, the Middle East, South Asia, and Latin America identify religion as “very important” in their lives, and the countries with the fastest growing populations are also highly religious (e.g., Niger, Uganda, Senegal, etc.) [4].

Robust scientific literature supports that individuals with greater religious involvement and religious/spiritual beliefs exhibit better physical health outcomes, including lower mortality risk [5,6,7,8,9]. For example, Li and colleagues found that weekly religious service attendance was associated with a 33% lower all-cause mortality risk compared to never-attenders among women in the Nurses’ Health study [5]. Additionally, these authors found that attending services once per week or more was related to a nearly five-fold decrease in the rate of suicide compared with never attending. For those attending less than once per week, these findings were partially mediated by depressive symptoms, social integration, and alcohol consumption, though the associations for those attending once per week or more were affected very little [10]. The aforementioned example is consistent with an earlier review of longitudinal studies that found a 30% reduction in the risk for mortality associated with regular religious service attendance [11].

Despite decades of evidence that regular religious service attendance is associated with healthier and longer lives, there are limited longitudinal data examining additional dimensions of religiousness and spirituality (R/S) in association with mortality. This oversight is important because there is considerable diversity in R/S, even among those who attend religious services [12,13,14,15]. For example, people of the same religious faith may relate differently to their faith and may attend religious services for different reasons. Noting this heterogeneity among religious individuals, Allport and Ross [13] developed the construct of religious orientation, positing that some individuals were intrinsically religious (i.e., their religious beliefs underlie their approach to life, religion is an end), whereas others were more extrinsically religiously orientated (i.e., religion serves as a means to other ends, such as social support, self-esteem, and/or personal comfort). Hood [16] expanded this into a four-fold typology, including intrinsic (intrinsic but not extrinsic), extrinsic (extrinsic but not intrinsic), pro-religious (both intrinsic and extrinsic), and non-religious (neither intrinsic nor extrinsic) orientations. Research demonstrates that these differences in religious orientation differentially predict mental and physical health outcomes [17,18,19]. The available evidence likewise suggests that additional aspects of R/S are linked with lower mortality risks. In the Black Women’s Health Study, positive religious/spiritual coping was associated with reduced mortality; however, associations were lessened after accounting for other religious/spiritual factors (religious service attendance, prayer, and religious/spiritual orientation) [20].

Behavioral, social, psychological, and physiological mechanisms are theorized to support the association between R/S and mortality [11,21]. Behavioral mechanisms are most often studied, and individuals who attend religious services regularly are less likely to be current smokers, consume less alcohol, are more likely to seek regular physical activity and are more likely to engage in preventive health behaviors (e.g., flu vaccination, cholesterol screenings) [1,22,23], although regular service attendance may also be related to overweight/obesity status [24]. In the Health and Retirement Study, Kim and VanderWeele examined multiple factors (i.e., positive psychological factors, psychological distress, health behaviors, social factors, and physical functioning) as mediators of the association between religious service attendance and mortality [6]. They found that greater life satisfaction, lower hopelessness and anger, greater contact with friends, greater exercise, and better physical functioning were each significant mediators. Nevertheless, further research using longitudinal study designs is needed to identify and test these relevant pathways.

In the current study, we focus on purpose in life and positive relations with others as potential mediators of the associations between religious service attendance and R/S with mortality. Purpose in life, referring to having goals and direction that contribute to having meaning in life, is particularly important in the context of religiousness/spirituality. Religion is a primary source from which many adults derive their meaning and purpose in life [25], and religious conversion has been shown to increase purpose in life as soon as one week after conversion [26]. Importantly, a robust literature supports that purpose in life is associated with lower mortality, and older adults with a high purpose in life had life expectancies at age 50 that were more than eight years longer than those with a low purpose in life [27,28]. The positive relations with others scale reflects the extent to which individuals have warm and trusting social relationships. Warm social connections may be cultivated within religious or spiritual communities and may provide both emotional and instrumental support to sustain health and longevity, including by buffering the deleterious effects of stress and promoting happiness and other positive emotions [29,30]. There may even be an upward spiral wherein happier people foster more positive social connections, which synergistically support health [31,32]. Social relationships are critically important for health and mortality, with one meta-analysis concluding that the effect sizes between social relationships and mortality were comparable to that of cigarette smoking and mortality [33].

In sum, the current study has two objectives. First, we examine the prospective associations between R/S, measured multidimensionally, and all-cause mortality in a national sample of midlife and older adults. We hypothesize that those who attend religious services regularly (i.e., at least weekly) will have a lower mortality risk compared to those who attend less regularly or not at all. Further, those who are higher on the additional dimensions of R/S (i.e., spirituality, religious identification, religious/spiritual coping) are predicted to have lower mortality risks, independent of sociodemographic and health factors. The second objective is to examine both purpose in life and positive relations with others, respectively, as potential mediators of the association between R/S and all-cause mortality. We hypothesize that purpose in life and positive relations with others will be significant mediators of the respective associations between R/S and all-cause mortality.

## 2. Materials and Methods

Data were collected from the core Midlife in the United States (MIDUS) survey sample. MIDUS began in 1995–1996 with a random-digit-dialing (RDD) of non-institutionalized adults in the 48 contiguous United States, siblings of the RDD respondents, a national sample of twins, and city-specific oversamples [34]. In 2004–2006, a longitudinal follow-up of the MIDUS 1 respondents was conducted (hereafter MIDUS 2). MIDUS respondents also provided a third wave of data in 2013–2015 (MIDUS 3). The response rate from the MIDUS 1 to MIDUS 2 surveys was 75%, adjusting for mortality [35], and the response rate from the MIDUS 2 to MIDUS 3 surveys was 77%, adjusting for mortality [36]. After consenting to the study, respondents completed a phone interview and self-administered questionnaire for each study wave. To be included in the current analyses, respondents had to provide complete demographic data (i.e., age, sex, race, education, marital status), baseline health status (i.e., count of chronic conditions) as well as complete the religiosity and spirituality items in the self-administered questionnaire. Compared with the participants with complete data (*N* = 6120), those with missing data (i.e., not included in the current analyses) were significantly younger (*t*(7103) = 8.59, *p* < 0.001), more likely to be male (Χ^2^ = 20.34, *p* < 0.001), less likely to be married or partnered (Χ^2^ = 111.97, *p* < 0.001), and had lower educational attainment (*t*(7093) = 8.35, *p* < 0.001).

### 2.1. All-Cause Mortality

Mortality data for the MIDUS respondents are available through December 2020. Decedents were identified via survey fielding, National Death Index searches, online tracing, and routine longitudinal sample maintenance [37]. A total of 1711 (28.0%) decedents were identified from the MIDUS 1 core sample with complete data. Respondents not identified as decedents (i.e., those presumed to be alive) were censored in December 2020.

### 2.2. Religiosity and Spirituality Measures

Service attendance was assessed with a single item that asked, “How often do you usually attend religious or spiritual services?” Five response options ranged from “never” to “more than once a week”.

Three scales of R/S were available among the MIDUS 1 respondents: religious/spiritual coping, spirituality, and religious identification [38]. Religious/spiritual coping was assessed with two items (e.g., “When you have decisions to make in your daily life, how often do you ask yourself what your religious or spiritual beliefs suggest you should do?”), and four response options ranging from “often” to “never”. In the analytic sample, internal consistency for religious/spiritual coping was α = 0.87. Spirituality was also assessed with two items (e.g., “How spiritual are you?”), and the internal consistency for spirituality was α = 0.91. Religious identification was assessed with six items (e.g., “How closely do you identify with being a member of your religious group?”), and the internal consistency was α = 0.89. The spirituality and religious identification items each had four response options, ranging from “very” to “not at all”. All scales were coded such that higher values reflected higher levels of religiousness/spirituality.

A principal components analysis showed that all items from the R/S coping, spirituality, and religious identification scales loaded highly on one factor (median factor loading = 0.78; range 0.66–0.85), which explained 59% of the variance. The Cronbach’s α for the composite was 0.92. Therefore, to minimize multiple comparisons, we created a R/S composite that averaged standardized (i.e., z-scored) R/S coping, spirituality, and religious identification measures into a single composite measure. When significant associations between the R/S composite and mortality were found, post hoc analyses examined the individual measures comprising the composite.

### 2.3. Purpose in Life and Positive Relations with Others

Purpose in life and positive relations with others were assessed using Ryff’s scales of psychological well-being [38,39]. In MIDUS 1, the purpose in life and positive relations with others scales included three items each, whereas, in MIDUS 2, the scales each included seven items. An example item for the purpose in life scale is “Some people wander aimlessly through life, but I am not one of them”. An example item for the positive relations with other scale is “People would describe me as a giving person, willing to share my time with others”. For both waves, the items had seven response options, ranging from “strongly agree” to “strongly disagree”. The internal consistency among the RDD sample for the purpose in life scale was 0.36 in MIDUS 1 (3 items) and 0.71 in MIDUS 2 (7 items). The internal consistency among the RDD sample for the positive relations with others scale was 0.58 in MIDUS 1 (3 items) and 0.77 in MIDUS 2 (7 items) [39]. The items were averaged to create scale scores for purpose in life and positive relations with others for MIDUS 1 and MIDUS 2, respectively, and the higher values reflect higher levels of purpose in life and positive relations with others. For mediation analyses, purpose in life and positive relations with others in MIDUS 2 were tested as mediators, with the respective scale in MIDUS 1 included as a covariate.

### 2.4. Covariates

Age, sex (1 = female, 0 = male), race (1 = white, 0 = other), educational attainment, marital status (1 = married or cohabitating, 0 = other) in MIDUS 1, and chronic conditions in MIDUS 1 were included as covariates in all models. Educational attainment was assessed with 12 categories, ranging from no schooling/some grade school to a doctoral or professional degree. Chronic conditions were assessed as a count of up to 29 conditions that respondents reported experiencing or being treated for in the prior 12 months.

### 2.5. Statistical Analysis

Cox regression models were used to estimate associations between religious service attendance and the religiousness/spirituality composite and all-cause mortality. Time to event was measured as the time between the MIDUS 1 interview date to the death date or censor. Separate models were run with either religious attendance or the religiousness/spirituality composite as the key predictors, and all models included age, sex, race, educational attainment, marital status, and chronic conditions from MIDUS 1. All continuous variables were mean-centered.

To examine the indirect effects of the religiousness/spirituality measures on mortality via purpose in life and positive relations with others, we calculated the indirect effects via the product of coefficients approach. To generate coefficients reflecting the associations between the predictors and mediators (i.e., ‘a’ paths), purpose in life and positive relations with others from MIDUS 2, respectively, were regressed on religious service attendance (coded to reflect at least weekly service attendance vs. less than weekly attendance) and the religiousness/spirituality composite in separate models. Age, sex, race, educational attainment, marital status, chronic conditions, and either purpose in life or positive relations with others from MIDUS 1 were included as covariates. The associations between the mediators and the outcomes (i.e., ‘b’ paths) were drawn from the Cox regression models that were identical to those described above, plus the addition of MIDUS 1’s purpose in life or positive relations with others. The PRODCLIN (i.e., distribution of the PRODuct Confidence Limits for Indirect effects) program calculated asymmetric confidence limits around the indirect effects (‘ab’ product term) [40]. Confidence limits that did not include 0 were interpreted as statistically significant.

## 3. Results

Table 1 displays descriptive information on the full analytic sample and separately by vital status. Compared to those who survived, those who died were older, less likely to be female, more likely to be white, less likely to be married or cohabitating, and more likely to have a high school education or less. Those who survived also had different service attendance patterns and had lower scores on the religiousness/spirituality composite, the religious/spiritual coping scale, and the religious identification scale compared to those who died. However, partial correlations holding the age constant showed that those who died were lower on all R/S measures than those who survived. Table A1 displays bivariate correlations among all study variables and partial correlations between vital status and R/S measures, holding age constant.

Cox regression models were used to examine associations between religious service attendance and the religiousness/spirituality composite and all-cause mortality. The results are presented in Table 2. Those who reported attending religious services about once per week or more than once per week had a lower risk of mortality compared to those who never attended religious services. Specifically, after controlling for age, sex, race, marital status, education, and chronic conditions, those who attended religious services multiple times per week had a 28% lower hazard rate (95% CI: 0.61, 0.85), and those who attended religious services approximately weekly had a 24% lower hazard rate (95% CI: 0.66, 0.88) compared to those who never attended religious services. However, there were no significant differences in mortality risk between those who attended religious services one to three times per month, or less than monthly, compared to never-attenders. The religiousness/spirituality composite was associated with a lower mortality risk as well, as were each of the individual scales comprising the composite. A one standard deviation increase in these religiousness/spirituality measures is associated with a 6–9% decrease in the hazard rate in models adjusting for age, sex, race, marital status, education, and chronic conditions.

### Indirect Effects via Purpose in Life and Positive Relations with Others

The product of coefficients approach was used to calculate the indirect effects from the R/S measures and mortality via purpose in life and positive relations with others [40]. Coefficients between the R/S measures and wave 2’s purpose in life and positive relations with others (i.e., ‘a’ paths), respectively, came from models that included age, sex, race, marital status, education, chronic conditions, and wave 1’s purpose in life or positive relations with others (Table 3). The R/S composite was positively associated with both purpose in life and positive relations with others, as were each of the variables comprising the composite. Those who reported attending religious services at least weekly also had significantly higher levels of both purpose in life and positive relations with others compared to those who reported attending services less than weekly. Coefficients between purpose in life and positive relations with others and mortality, respectively, (i.e., ‘b’ paths) were drawn from Cox regression models that included age, sex, race, marital status, education, chronic conditions, purpose in life or positive relations with others from wave 1, and the R/S measures (each R/S measure was assessed in a separate model).

Table 4 presents the indirect effects and asymmetric confidence intervals for the mediation results (i.e., a × b paths). In all cases, the indirect effects from each of the R/S measures on mortality via purpose in life or via positive relations with others were significantly different from zero (i.e., zero was not contained in the confidence limits). As such, the results support both purpose in life and positive relations with others as statistical mediators of the protective associations between R/S and mortality. The direct effects from the R/S composite, R/S coping, spirituality, or religious identification to mortality with either purpose in life or positive relations with others in the model were not significantly different from zero. The direct effects from weekly service attendance to mortality with either purpose in life or positive relations with others in the model were significantly different from zero (HRs = 0.83, 95% CIs [0.73, 0.95]).

Given the low internal consistencies of the purpose in life and positive relations with others scales in MIDUS 1, sensitivity analyses were run that included an 18-item psychological well-being composite in MIDUS 1 (internal consistency = 0.81; [38]) as a covariate instead of the 3-item purpose in life or positive relations with others scales, respectively. Conclusions from the sensitivity analyses are identical to those reported above.

## 4. Discussion

A robust scientific literature, over recent decades, links more frequent religious service attendance to lower mortality [1,11]. In this study, we demonstrated that additional dimensions of R/S were also associated with lower mortality risk, as was weekly (or more) service attendance compared to never attending services. These additional aspects of R/S are important to study because they expand the discussion around how involvement with and experiences of the religious and spiritual aspects of life might also affect health and mortality. Though differences in the frequency of service attendance capture one critical aspect of religious behavior, many other dimensions of R/S are needed to fully characterize the phenomenological or experiential heterogeneity among religious or spiritual involvement, practices, salience, and beliefs. The results showed that R/S coping, spirituality, and religious identification were collectively and independently associated with lower mortality risks over a 25-year follow-up (1995–2020).

The findings regarding these various dimensions of R/S experience are important in helping to better understand the robust finding, replicated here, that religious service attendance predicts lower mortality risk. Though this is a replicated and stable empirical result, religious service attendance is lacking as an explanatory variable regarding mortality. Surely, it is not simply the act of presenting oneself at a religious service that is efficacious. What is it about attendance at religious services that predict health and mortality? Are there particular psycho-religious variables that offer greater illumination regarding these relationships? The three scales employed in our study, named: (a) religious/spiritual coping, (b) spirituality, and (c) religious identification, shed some light on this issue. Taken together, these scales investigate the importance or value of religion and spirituality in individuals’ lives, the application of religious and spiritual perspectives to solve problems and find comfort, and the strength of the communal identity that individuals have toward their religious group. These R/S dimensions may support health and longevity by promoting psychological well-being, supporting strong social connections, buffering against stress, and encouraging healthy behaviors. Though these psycho-religious measures may be cultivated via attendance at religious services, they may also be fostered independently of service attendance. Indeed, prior research in the MIDUS study found that spiritual perceptions were a stronger predictor of psychological well-being than service attendance when both were entered as simultaneous predictors [41].

The other primary objective of the study was to examine two psychological mechanisms that may explain why R/S is associated with lower mortality in the population. Specifically, we focused on purpose in life, or the extent to which individuals are goal-directed and have meaning in their lives, and positive relations with others, which captures the presence of close, trusting social relationships as possible mediators. The results showed that both purpose in life and positive relations with others were significant pathways through which each of the R/S measures was associated with mortality. Though psychological and social mechanisms are often posited as relevant mechanisms explaining why religiousness and spirituality are associated with health and mortality outcomes [21,42], this study is unique because it allowed for an empirical test of such pathways given the data on multiple dimensions of R/S, high-quality measurements of purpose in life and positive relations with others, and a longitudinal study design among a large, diverse sample of adults. Importantly, the pathway from R/S to mortality via purpose in life is not necessarily independent of the pathway via positive relations with others. Purpose in life and positive relations with others are correlated constructs, and many adults derive purpose in life from their social relationships [43]. It is also possible that those with greater purpose demonstrate better emotional stability and positive affect, which likely contribute to more positive social relationships. In fact, changes in social support and strain are correlated with changes in purpose in life over time among older adults [44]. Future research with more intensive measurement designs should interrogate the reinforcing connections between various aspects of R/S and involvement with R/S communities, purpose in life, and the development and maintenance of warm, trusting social connections over time.

Prior research among older adults in the Health and Retirement Study (HRS) found that higher life satisfaction and more frequent contact with friends, in addition to lower hopelessness, anger, and loneliness, were significant mediators of the association between weekly service attendance and mortality. However, purpose in life or other social factors (e.g., marital status, contact with children or other family members) did not emerge as significant mediators [6]. Purpose in life declines sharply in older ages, perhaps due to role changes (e.g., retirement) [45,46], and correlations between purpose in life and social support and loneliness are weaker among older adults relative to emerging and midlife adults [47]. Taken together, it may be that purpose in life is most relevant as a mediator of R/S and mortality links in midlife (i.e., the MIDUS sample) as compared to older ages (i.e., the HRS sample). Regarding the discordant findings between MIDUS and HRS related to positive social support, the measure of positive social support included in the current study specifically focused on capturing empathy, promoting the welfare of others, and cultivating satisfying relationships [48]. Thus, this measure is distinct from the frequency of contact with friends, children, and other family captured in the HRS [6]. Religiousness and spirituality are thought to promote both emotional and instrumental aspects of social support that are important for health and mortality [49], and this may be better captured with a more psychologically oriented measure of positive social support, such as the measure used in the current research. Future research should pursue psychological mediators that promote health and longevity in the context of R/S and consider whether these associations are consistent across the life-course.

Though we were able to expand the longitudinal investigation of R/S and mortality beyond religious service attendance by including an assessment of other aspects of religious/spiritual life, we were constrained by the variables available in MIDUS and acknowledge that many other potentially important R/S constructs remain to be studied. Further, we suspect that not all of these constructs will demonstrate the type of beneficial associations that we found. For example, psychological well-being may decline when religion is imposed or supported by extrinsic (vs. intrinsic) motivations [21,50]. Relatedly, the use of R/S for coping can either support or impede well-being, depending on the type of coping methods used. Positive R/S coping occurs when an individual has a secure relationship with a transcendent force and uses this relationship to cope with challenges [51]. This type of coping results in positive health and well-being outcomes [20]. On the other hand, negative R/S coping occurs when an individual has spiritual tensions within oneself, others, and the divine, such as feeling punished or abandoned by God when a challenge arises. This type of coping often predicts adverse health outcomes [52]. We likewise did not examine the precursors to R/S, such as personality and genetic contributions, which may account for some of the association between R/S and mortality. Investigators should continue moving toward the use of multidimensional measures of R/S, including personality and genetic precursors, in research to better understand the nuances in R/S that may affect health and well-being [49].

Additional limitations warrant consideration. First, the MIDUS sample underrepresented racial/ethnic minorities, and those included in the analytic sample were different demographically than the full sample, which may affect the generalizability of the results. Second, causal claims regarding R/S, purpose in life, positive relations with others and mortality are not warranted, given the observational study design, although we did utilize the longitudinal data available in MIDUS (i.e., the predictors were measured at wave 1 and the mediators were measured at wave 2). Third, the internal consistencies of purpose in life and positive relations with others at wave 1 were low, although sensitivity analyses suggest that the mediation results were robust to this limitation. Finally, given the recent declines in religious service attendance [2], it is important to emphasize that the data reported herein reflect associations between the R/S assessed in the mid-1990s with prospective mortality. Future research is needed to examine whether and how R/S affects health and mortality in future cohorts. Notwithstanding these limitations, this study adds important evidence about the key dimensions of R/S that are relevant for mortality and identifies purpose in life and positive relations with others as psychological mechanisms underlying salubrious associations between R/S and mortality in a large, diverse sample of midlife and older adults.

## 5. Conclusions

This study replicates and extends prior research on R/S as being predictive of lower mortality. In a large sample of midlife and older adults, attending religious services at least weekly was associated with lower mortality compared to never-attenders. Further, other key aspects of R/S were also associated with lower mortality, suggesting that spirituality, religious beliefs, and using religion/spirituality to cope with stress also support longer lives. Importantly, purpose in life and positive relations with others were statistical mediators of the associations between R/S and mortality. These psychological mechanisms may be fostered among religious or spiritual communities and may play important roles in maintaining health across a life’s course. This research on the processes that help explain why those with religious and spiritual affiliations and beliefs live healthier and longer lives is needed, given the centrality of religious and spiritual beliefs in guiding cognitions, emotions, behaviors, and interpersonal functioning for large segments of the population.

## Figures and Tables

**Table 1:** Descriptive information on the analytic sample.

Variable ^1^	Full Sample	Survived	Died
Sample size	6120	4409	1711
Age, in years ^2^	46.9 (12.9)	42.3 (10.5)	58.9 (10.6)
Sex (% female) ^2^	52.7% (3223)	54.0% (2397)	48.3% (826)
Race (% White) ^2^	90.7% (5553)	90.1% (3974)	92.3% (1579)
Education ^2^ (% ≤ high school education)	37.3% (2280)	33.1% (1458)	48.0% (822)
Marital status ^2^ (% married/cohabitating)	68.0% (4164)	69.1% (3048)	65.2% (1116)
Chronic conditions, count ^2^	2.4 (2.5)	2.1 (2.3)	3.2 (2.9)
Religious service attendance ^2^			
% more than weekly	12.7% (780)	11.9% (525)	14.9% (255)
% weekly	26.0% (1593)	25.5% (1125)	27.4% (468)
% one to three times/month	13.3% (812)	13.8% (610)	11.8% (202)
% less than monthly	27.9% (1710)	29.0% (1277)	25.3% (433)
% never	20.0% (1225)	19.8% (872)	20.6% (353)
Religiousness/spirituality composite ^2^	0.0 (0.9)	−0.02 (0.9)	0.05 (0.9)
Religious/spiritual coping ^2^	2.7 (1.1)	2.7 (1.1)	2.8 (1.1)
Spirituality	3.1 (0.8)	3.1 (0.8)	3.1 (0.8)
Religious identification ^2^	2.8 (0.8)	2.7 (0.8)	2.8 (0.7)
Purpose in life, wave 1 ^2^	5.5 (1.2)	5.6 (1.1)	5.2 (1.3)
Purpose in life, wave 2 ^2^	5.5 (1.0)	5.6 (1.0)	5.2 (1.0)
Positive relations with others, wave 1 ^2^	5.4 (1.4)	5.4 (1.3)	5.3 (1.4)
Positive relations with others, wave 2	5.8 (1.0)	5.8 (1.0)	5.8 (1.0)

^1^ Data are presented as Mean (Standard Deviation) or % (n). ^2^ Denotes significant difference by vital status, *p* < 0.05.

**Table 2:** Cox regression results for religiousness and spirituality measures predicting mortality.

Variable	B(SE)	*p*	HR [95% CI]
Age ^1^	0.10 (0.002)	<0.001	1.10 [1.10, 1.11]
Sex (1 = female) ^1^	−0.46 (0.05)	<0.001	0.63 [0.57, 0.70]
Race (1 = white) ^1^	−0.04 (0.09)	0.63	0.96 [0.80, 1.15]
Marital status (1 = married/partnered) ^1^	−0.29 (0.05)	<0.001	0.75 [0.68, 0.83]
Education ^1^	−0.07 (0.01)	<0.001	0.93 [0.91, 0.95]
Chronic conditions ^1^	0.08 (0.01)	<0.001	1.09 [1.07, 1.11]
Religiousness/spirituality composite	−0.09 (0.03)	0.001	0.91 [0.86, 0.97]
Religious/spiritual coping	−0.07 (0.03)	0.007	0.93 [0.89, 0.98]
Spirituality	−0.07 (0.03)	0.009	0.94 [0.89, 0.98]
Religious identification	−0.08 (0.03)	0.002	0.93 [0.88, 0.97]
Religious service attendance (ref = never)			
More than weekly	−0.33 (0.09)	<0.001	0.72 [0.61, 0.85]
Approximately weekly	−0.27 (0.07)	<0.001	0.76 [0.66, 0.88]
One to three times per month	−0.15 (0.09)	0.098	0.86 [0.72, 1.03]
Less than monthly	−0.09 (0.07)	0.21	0.91 [0.79, 1.05]

^1^ Reported coefficients for covariates are from a model containing no religiousness or spirituality variables.

**Table 3:** Ordinary least squares regression results for religiousness and spirituality measures predicting wave 2’s purpose in life and positive social support.

	DV = Purpose in Life, Wave 2		DV = Positive Relations with Others, Wave 2	
Variable	B(SE)	*p*	B(SE)	*p*
Age ^1^	0.01 (0.01)	0.40	0.05 (0.01)	<0.001
Sex (1 = female) ^1^	0.65 (0.21)	0.002	1.14 (0.19)	<0.001
Race (1 = white) ^1^	−0.16 (0.42)	0.71	0.40 (0.38)	0.30
Marital status (1 = married/partnered) ^1^	1.08 (0.23)	<0.001	0.48 (0.21)	0.024
Education ^1^	0.30 (0.04)	<0.001	0.10 (0.04)	0.011
Chronic conditions ^1^	−0.35 (0.04)	<0.001	−0.18 (0.04)	<0.001
Purpose in life, wave 1 ^1^	2.23 (0.09)	<0.001		
Positive relations with others, wave 1 ^1^			2.72 (0.07)	<0.001
Religiousness/spirituality composite	0.93 (0.12)	<0.001	0.66 (0.11)	<0.001
Religious/spiritual coping	0.57 (0.10)	<0.001	0.37 (0.10)	<0.001
Spirituality	0.86 (0.10)	<0.001	0.61 (0.10)	<0.001
Religious identification	0.67 (0.10)	<0.001	0.51 (0.10)	<0.001
≥Weekly religious service attendance (vs. <weekly)	0.73 (0.21)	<0.001	0.54 (0.19)	0.006

*N* = 3284. ^1^ Reported coefficients for covariates are from a model containing no religiousness or spirituality variables. Each R/S variable was entered into a separate regression model.

**Table 4:** Indirect effects from wave 1’s religiousness and spirituality measures to mortality via wave 2’s purpose in life and positive relations with others.

	Estimate	95% CI
**Variable** **→ Purpose in Life** **→ Mortality**		
Religiousness/spirituality composite	−0.14	−0.23, −0.06
Religious/spiritual coping	−0.09	−0.15, −0.04
Spirituality	−0.13	−0.21, −0.06
Religious identification	−0.10	−0.17, −0.04
≥Weekly religious service attendance (vs. <weekly)	−0.11	−0.21, −0.04
**Variable** **→ Positive Relations with Others** **→ Mortality**		
Religiousness/spirituality composite	−0.08	−0.14, −0.03
Religious/spiritual coping	−0.04	−0.09, −0.01
Spirituality	−0.07	−0.13, −0.02
Religious identification	−0.06	−0.11, −0.02
≥Weekly religious service attendance (vs. <weekly)	−0.06	−0.14, −0.01

*N* = 3284. Models included age, sex, race, marital status, education, chronic conditions, and either wave 1 purpose in life or positive relations with others.

## Data Availability

Data and documentation for the MIDUS study can be found at https://www.icpsr.umich.edu/web/ICPSR/series/203 (accessed on 30 September 2022).

## Appendix A

**Table A1:** Bivariate correlations among study variables.

	2.	3.	4.	5.	6.	7.	8.	9.	10.	11.	12.	13.	14.
1. Vital status (1 = died)	0.04 **	0.04 **	−0.00	0.06 ***	0.05 ***	0.58 ***	−0.06 ***	0.03 **	−0.16 ***	−0.04 **	0.20 ***	−0.17 ***	−0.05 ***
2. Religiousness/spirituality composite		0.90 ***	0.87 ***	0.87 ***	0.60 ***	0.13 ***	0.23 ***	−0.10 ***	−0.01	0.03 **	0.06 ***	0.04 ***	0.14 ***
3. Religious/spiritual coping			0.68 ***	0.68 ***	0.52 ***	0.13 ***	0.24 ***	−0.06 ***	0.01	0.01	0.07 ***	0.03 **	0.11 ***
4. Spirituality				0.60 ***	0.44 ***	0.07 ***	0.21 ***	−0.09 ***	0.02	−0.01	0.05 ***	0.05 ***	0.14 ***
5. Religious identification					0.62 ***	0.15 ***	0.16 ***	−0.10 ***	−0.05 ***	0.08 ***	0.03 *	0.03 *	0.12 ***
6. ≥Weekly religious service attendance						0.17 ***	0.09 ***	−0.03 *	0.04 ***	0.12 ***	−0.00	0.03 *	0.12 ***
7. Age							0.01	0.10 ***	−0.11 ***	0.05 ***	0.18 ***	−0.15 ***	0.04 **
8. Female sex (vs. male sex)								−0.03 *	−0.09 ***	−0.11 ***	0.12 ***	−0.04 **	0.10 ***
9. White race (vs. other race)									0.05 ***	0.12 ***	−0.02	0.03 *	0.03 *
10. Education										0.00	−0.13 ***	0.25 ***	0.08 ***
11. Married or cohabitating (vs. not)											−0.09 ***	0.14 ***	0.18 ***
12. Chronic conditions												−0.19 ***	−0.18 ***
13. Purpose in life, wave 1													0.37 ***
14. Positive relations with others, wave 1													

Note. N = 6120. *** *p* < 0.001, ** *p* < 0.01, * *p* < 0.05. Partial correlations holding age constant showed that vital status (1 = died) was inversely correlated with all religiousness/spirituality (R/S) measures (R/S composite *pr* = −0.05, *p* < 0.001; R/S coping *pr* = −0.05, *p* < 0.001; Spirituality *pr* = −0.05, *p* < 0.001; R/S identification *pr* = −0.03, *p* = 0.008; Weekly service attendance *pr* = −0.06, *p* < 0.001).
